# Impact of Cyclosporine Levels on the Development of Acute Graft versus Host Disease after Reduced Intensity Conditioning Allogeneic Stem Cell Transplantation

**DOI:** 10.1155/2014/620682

**Published:** 2014-01-30

**Authors:** Irene García Cadenas, David Valcarcel, Rodrigo Martino, J. L. Piñana, Pere Barba, Silvana Novelli, Albert Esquirol, Ana Garrido, Silvana Saavedra, Miquel Granell, Carol Moreno, Javier Briones, Salut Brunet, Jorge Sierra

**Affiliations:** ^1^Hematology Department, Hospital de la Santa Creu i Sant Pau, Universitat Autònoma de Barcelona, Banc de Sang i Teixits, Institut Josep Carreras, Mas Casanovas 90, 08025 Barcelona, Spain; ^2^Hematology Department, Hospital Vall d'Hebron, Universitat Autònoma de Barcelona, Barcelona, Spain; ^3^Hematology Department, Hospital Clínic de Valencia, Valencia, Spain

## Abstract

We analyze the impact of cyclosporine (CsA) levels in the development of acute graft-versus-host disease (aGVHD) after reduced intensity conditioning allogeneic hematopoietic transplantation (allo-RIC). We retrospectively evaluated 156 consecutive patients who underwent HLA-identical sibling allo-RIC at our institution. CsA median blood levels in the 1st, 2nd, 3rd and 4th weeks after allo-RIC were 134 (range: 10–444), 219 (54–656), 253 (53–910) and 224 (30–699) ng/mL; 60%, 16%, 11% and 17% of the patients had median CsA blood levels below 150 ng/mL during these weeks. 53 patients developed grade 2–4 aGVHD for a cumulative incidence of 45% (95% CI 34–50%) at a median of 42 days. Low CsA levels on the 3rd week and sex-mismatch were associated with the development of GVHD. Risk factors for 1-year NRM and OS were advanced disease status (HR: 2.2, *P* = 0.02) and development of grade 2–4 aGVHD (HR: 2.5, *P* < 0.01), while there was a trend for higher NRM in patients with a low median CsA concentration on the 3rd week (*P* = 0.06). These results emphasize the relevance of sustaining adequate levels of blood CsA by close monitoring and dose adjustments, particularly when engraftment becomes evident. CsA adequate management will impact on long-term outcomes in the allo-RIC setting.

## 1. Introduction

Myeloablative allogeneic hematopoietic cell transplantation is the standard of care therapy for patients with several hematologic malignancies, but its high treatment-related mortality (TRM) frequently counterbalances its beneficial effects. Currently, reduced intensity conditioning (RIC) allows allogeneic transplantation in patients otherwise considered ineligible because of advanced age or associated comorbidities. Although early TRM is reduced with RIC regimens, the development of graft-versus-host disease (GVHD) remains an important cause of transplant related morbidity and mortality [1–3].

Posttransplantation administration of immunosuppressive agents remains the most widely used strategy to prevent GVHD. A calcineurin inhibitor, mainly Cyclosporine-A (CsA) in combination with a second drug, remains the most common approach to prevent the occurrence of GVHD [4].

Several studies on myeloablative conditioning allogeneic transplantation have shown that low whole blood concentrations of CsA during the periengraftment period can strongly affect the incidence of grade 2–4 acute GVHD (aGVHD) [5, 6]. The impact of CsA levels in allo-RIC setting has been less studied and justified the investigation reported here. Accordingly, this study evaluated the impact of CsA levels on the development of moderate to severe aGVHD, TRM, and overall survival (OS) in a cohort of consecutive HLA-identical sibling allo-RIC recipients.

## 2. Patients and Methods

### 2.1. Patients

We analyzed the data of 156 consecutive adult patients included in two prospective allo-RIC cohorts from our center transplanted between April 1999 and January 2010. All patients were diagnosed with hematological malignancies and received granulocyte-colony stimulating factor (G-CSF-) mobilized peripheral blood stem cells from HLA-identical siblings without *in vivo*/*ex vivo* T-cell depletion. HLA matching was performed by low resolution techniques for HLA A and HLA B and at the allelic level for HLA-DRB1 [7]. All participants gave written informed consent and the studies were approved by the national and local ethics committees.

### 2.2. Transplant Conditioning and GVHD Prophylaxis

The conditioning regimen consisted of intravenous fludarabine 150 mg/m^2^ or its equivalent oral doses (200 mg/m^2^) combined with either targeted doses of oral busulfan 10 mg/kg (8 mg/kg for patients >65 years) for myeloid malignancies or melphalan 140 mg/m^2^ (70 mg/m^2^ for patients >65 years) for lymphoid malignancies. GVHD prophylaxis administered was CsA plus either mycophenolate mofetil (MMF) or short course methotrexate (MTX). CsA was started on day −7 in most patients and administered at an initial dose of 1.5 mg/kg/12 h as a 2-hour infusion and then adjusted to maintain blood levels of 200–300 ng/mL. CsA was switched to an oral formulation at a ratio of 1 : 1 when patients were able to tolerate oral intake.

From 1999 to 2003, MTX was administered on days +1, +3, and +6 (10 mg/m^2^) and folinic acid rescue was administered 24 h after each dose (*n* = 121, 78%). In 2004 MTX was substituted for MMF (*n* = 35, 22%) in an effort to reduce MTX-related toxicity. MMF was started on day 0 (at least 10 h after the infusion of progenitors) at a dose of 15 mg/kg/8 h and continued until day +30, when it was tapered in the absence of aGVHD. As we have described previously [8, 9] and confirmed again in our patient population, the use of CsA+MTX versus CsA+MMF or conditioning with busulphan versus melphalan had no impact on the occurrence of grade 2–4 aGHVD; so we analyzed all the patients as a whole group.

### 2.3. Cyclosporine Dose Adjustment and GVHD Assessment

CsA whole-blood concentration was measured by radioimmunoassay [10] at least twice weekly during the first four weeks after transplantation. Blood samples were collected 12 hours after the prior dose, immediately before the morning dose. According to our current practice, when levels were between 100 and 200 ng/mL the dose was increased by 25%. If CsA concentrations were below 100 ng/mL and there was no renal impairment the dose was increased by 50%. When the blood concentrations were between 301 and 450 or 451 and 600 ng/mL the dose was decreased by 25% and 50%, respectively. If blood concentrations exceeded 601 ng/mL the next dose was omitted and the drug was restarted at half the dose. Renal and liver function and electrolyte concentrations were monitored daily during admission and at outpatient visits. When kidney impairment occurred (defined as a decrease of >25% of base-line glomerular filtration rate (GFR)) the CsA dose was decreased by 25%. If there were no changes in renal function, the drug was maintained in reduced doses and administered as a 24 h continuous infusion. When severe renal failure occurred (defined as decrease in GFR >50%), CsA was stopped until the renal function recovered, irrespective of the CsA blood levels.

CsA was also discontinued in case of severe thrombotic microangiopathy, defined as the presence of 2 or more schistocytes per high power field on peripheral smear with concurrent increased serum lactate dehydrogenase and renal or neurological dysfunction without other explanations or positive direct Coombs' test result.

Whenever CsA was stopped due to any severe complication, steroids were introduced at a dose of 2 mg/kg/day of prednisone. The dose of steroids was maintained or slowly tapered until the complications resolved or markedly improved; CsA was then restarted at a lower dose, or another immunosuppressive drug was initiated if CsA could not be reinitiated (MMF in most cases).

Diagnosis of aGVHD was based on clinical findings and confirmed with histological evaluation of affected organ(s). The overall grading followed the Przepiorka standard criteria [11]. Anti-infectious prophylaxis was performed with acyclovir, quinolones (norfloxacin or ciprofloxacin), and fluconazole. Trimethoprim-sulfamethoxazole or nebulized pentamidine were also used as prophylaxis for at least six months. G-CSF was not routinely administered. Serial serum monitoring of Aspergillus galactomannan was performed in all patients since 2004 [12]. A preemptive strategy against cytomegalovirus guided by antigenemia or PCR was performed, as described elsewhere in details [13].

### 2.4. Risk Assessment

Advanced disease status was defined as acute leukemia in ≥2nd complete response (CR), myeloproliferative disease in accelerated/blast phase or ≥2nd CR, Hodgkin's disease and follicular lymphoma in ≥3rd CR, and large B-cell lymphoma or multiple myeloma in ≥2nd CR. Patients with partial response (PR) or persistent disease at transplantation (except for MM) were also considered as advanced disease status.

### 2.5. Statistical Considerations

The primary endpoint of the study was to assess the effect of Cyclosporine concentrations on the development of 2–4 aGVHD. The median concentration of CsA blood levels during a given week was calculated for each patient using the different concentrations obtained during that week, as previously reported [5, 14]. The median CsA in different weeks was compared with the ANOVA tests. The impact of CsA levels on posttransplant outcomes was calculated treating this variable as a time-dependent covariate with the event occurring at the onset of aGVHD. Overall survival (OS) and the cumulative incidence of nonrelapse mortality were secondary endpoints.

Univariate analyses of the association of various clinical risk factors with posttransplantation outcomes were calculated using univariate Cox regression models, whereas the log-rank test was used for OS. Multivariate analyses were performed by Cox proportional hazards regression, and variables with a *P* value < 0.1 in the univariate testing were included. *P* values < 0.05 were considered statistically significant, and the hazard ratios (HRs) and their 95% confidence intervals (95% CIs) were calculated. The assumption of proportional hazards over time was tested for all explanatory covariates by using a time-dependent covariate and the analysis of aGVHD and NRM were calculated taking into account relapse as a competing event [15–17]. Survival was estimated by the Kaplan-Meier method, and comparisons of actuarial curves were made with the log-rank test. All statistical analyses were performed using SPSS version 19.0 with the exception of the cumulative incidence analyses which were performed with NCSS 2004 (Number Cruncher Statistical System, Kaysville, UT).

## 3. Results

### 3.1. Patient and Transplant Characteristics

Characteristics of the patients are shown in detail on Table 1. The main reasons for receiving RIC instead of myeloablative conditioning were age >55 years (*n* = 77, 49%), multiple prior therapy lines or autologous HCT (*n* = 24, 15%), and significant medical comorbidities (*n* = 10, 6%). Forty-five patients (29%) presented more than one of the previous circumstances. Fifty-three patients with a myeloid malignancy (35%) received fludarabine-busulfan conditioning, while the 103 patients (65%) with lymphoid malignancies received fludarabine-melphalan. The median follow-up of survivors was 67 (5–121) months.

### 3.2. Cyclosporine Levels

Table 2 shows in details the results of the CsA levels during the first 4 weeks after allo-RIC. The median blood concentration of CsA in the 1st week after transplantation was lower than in the 2nd, 3rd, and 4th weeks, with values of 134 (range: 10–444), 219 (range: 54–656), 253 (range: 53–910), and 224 (range: 30–699) ng/mL, respectively (*P* < 0.001). Eighty-nine (60%), 24 (16%), 16 (11%), and 21 (17%) patients had median CsA levels below 150 ng/mL during these weeks. Only 15% of patients had CsA levels within the “optimal” range of 200–300 ng/mL in the first week, while around 45% of the cases had a median level within this optimal range from the 2nd to the 4th week after transplantation.

In our series 77 patients began CsA on day −7. We did not find significant differences in CsA levels during the first week between this group and those who started CsA in day −1, but the number of patients is small, and these results would need a proper study.

### 3.3. Incidence and Risk Factors for Acute Graft versus Host Disease

**Figure 1:**
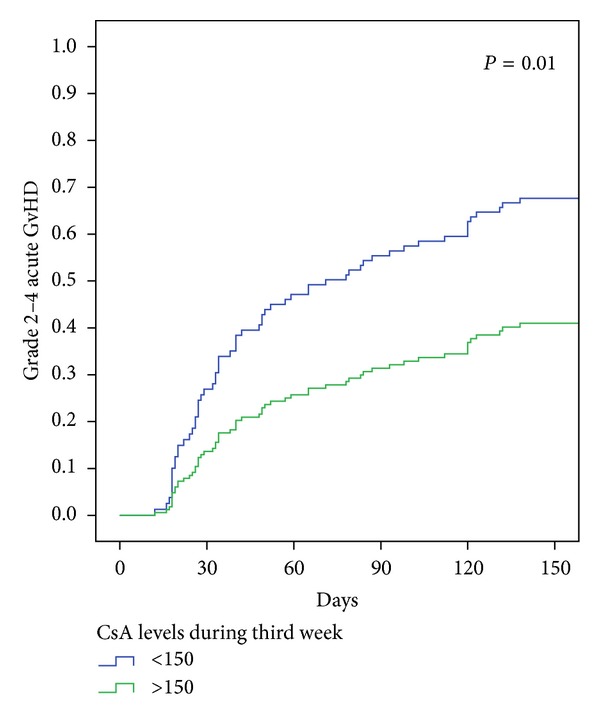
Cumulative incidence of grades 2–4 aGVHD. Cumulative incidence of grades 2–4 aGVHD depending on CsA concentration during the third week posttransplantation. The patients with median CsA levels lower than 150 ng/mL are included in the group “Low CsA levels.” CsA: cyclosporine; aGVHD: acute graft versus host disease.

55 patients (36%) developed 2–4 aGVHD for a cunulative incedence of 45% (34–50%) at day +180. aGVHD appeared at a median time of 42 (range: 16–185) days after allo-RIC. Further details of aGVHD are shown in Table 3. The median CsA blood concentrations in the second and third weeks after transplantation were lower in patients who developed grade 2–4 aGVHD than in the remainder (201 versus 237 ng/mL, *P* = 0.01, and 248 versus 283 ng/mL, *P* = 0.06 shown in Figure 1); in contrast, there were no significant differences in the levels when analyzed on the first (143 versus 139, *P* = 0.7) and fourth weeks (226 versus 254, *P* = 0.2). In univariate analysis the variables associated with a higher incidence of grade 2–4 aGVHD were sex-mismatch (female donor to male recipient versus other combinations (*P* < 0.01)) and low median CsA blood levels (defined as median CsA levels below 150 ng/mL) in the second (*P* = 0.02) and third (*P* = 0.01) weeks after allo-RIC. CMV status, disease phase at SCT, age of the recipient, donor age, conditioning (busulphan versus melphalan), GVHD prophylaxis with MMF versus MTX, and CsA levels in the first and fourth weeks after transplantation were not associated with aGVHD (detailed in Table 4). In multivariate analysis the only significant variables associated with grade 2–4 aGVHD were low CsA concentration during the third week after transplantation and female-to-male sex-mismatch, as shown in Table 4.

### 3.4. Toxicity and Early Discontinuation of Cyclosporine

Renal impairment occurred in 49 patients (31%) during the first 5 weeks after transplantation. CsA was the only cause of renal failure in 35 of these patients (71%). Six patients (3.8%) developed a thrombotic microangiopathy (TMA). The median concentration in the 7 days before the onset of renal failure or TMA did not differ in patients with CsA-related toxicity and those with other causes of renal dysfunction (244 versus 211, resp.; *P* = 0.2). The median CsA during the first month in patients without renal impairment was 205 ng/mL (range 92–457) compared to 208 ng/mL in those patients who presented nephrotoxicity (range: 10–611; *P* = 0.4).

CsA blood concentration was higher than 300 ng/mL in only 9/49 (18%) cases with renal impairment. Interestingly there were not differences in the incidence of aGVHD between patients who developed nephrotoxicity and those without renal impairment (*P* = 0.4).

### 3.5. Nonrelapse Mortality and Overall Survival

Table 5 shows the variables identified as risk factors for the 1-year NRM and OS in univariate and multivariate analyses. The cumulative incidence of NRM for the whole group at +180 days and 1 year was 15% (95% CI: 11–22) and 20% (95% CI: 15–28), respectively. In the multivariate analysis the variables found to have a negative impact on NRM were advanced disease status at transplantation (HR 2.2, *P*: 0.01) and development of grade 2–4 aGVHD (HR: 2.5, *P*: 0.01), whereas low median CsA blood levels on the third week after transplantation showed a trend as additional independent risk factor for NRM (*P*: 0.06).

The 1-year and 5-year probability of OS were 67% (95% CI: 59–74.5%) and 45% (95% CI: 33–51%), respectively. Relapse (39%) was the main cause of death, followed by GVHD (aGVHD: 14 (16%), chronic GVHD: 10 (11.5%)), and infectious complications (*n* = 16, 18%). The median time to death from any cause was 7.8 months (range: 0.2–94 months). In multivariate analysis, the only two variables associated to adverse OS were advanced disease status at SCT (HR: 2.2, *P*: 0.02) and development of grade 2–4 aGVHD (HR 2.5, *P* < 0.01).

## 4. Discussion

The current study found that, despite close surveillance, the median CsA levels were outside the desired range in a proportion of patients during the early posttransplant period. Of note, low median levels of CsA during the third week post-SCT were associated with a higher risk of grade 2–4 aGVHD and a trend towards increased risk of NRM.

Despite significant improvements in terms of therapeutic drug monitoring in the last years, one of the most important and unexpected finding in the current study was the substantial proportion of patients with low levels of CsA in the first weeks post-SCT. Despite continuous changes in the dose of CsA, approximately 15% of the patients maintained low levels of CsA during the following three weeks.

Possible explanations for these findings could be the use of short perfusion of CsA, compared to the 8 or 24 h continuous infusion administered in other centers, a less stringent monitoring of CsA levels (every other day in Song et al.'s study) [14, 18, 19] or a conservative approach when CsA dose had to be increased in those patients with very low CsA previous concentrations. In our series, there appears to be no benefit in starting CsA on day −7 instead of −1, supporting the results by Lanino et al. [20].

Renal impairment is a well-known potential toxicity of CsA that appears to be dose related and reversible [21, 22]. The 31% rate of renal impairment during the first month post-SCT in this cohort of patients is similar to previous reports [23, 24]. CsA was considered responsible of renal function impairment in 71% of the cases. Surprisingly, there were no differences in the median CsA concentration the week prior to the onset of renal failure compared to the blood levels in patients who maintained a normal kidney function, even in the 71% of patients who were considered to have CsA-related renal failure.

The incidence of grade 2–4 aGVHD in our population (45% (95% CI 34–50%)) was similar to previous studies in the allo-RIC setting [25] and it was statistically significantly higher in patients with low median CsA levels during the third week post-SCT. This finding may be due to the need of having therapeutic levels of CsA especially during the time of lymphohematopoietic recovery, which was around day +15 (range: 10–29) in our series. This finding is similar to a recently published study analyzing the impact of tacrolimus levels after stem cell infusion (OR: 0.76, 95% CI: 0.58–0.98, *P* < 0.05 for those patients with high concentrations of the calcineurin inhibitor in the third week post-SCT) [26]. Other studies have shown the relationship between low CsA concentrations and increased risk of aGVHD, but conflicting findings about the moment in which this association is more important have been reported. A recent publication by Malard et al. [6], analyzing a series of patients receiving both conventional and RIC regimens, found that the CsA blood levels in the first week after graft infusion was the strongest risk factor for severe aGVHD (*P* = 0.012, RR = 0.24).

Although the development of RIC regimens has allowed patients who are ineligible for standard Allo-SCT to potentially benefit from allogeneic therapy, NRM remains a significant obstacle to the success of Allo-RIC. Our study found that advanced disease status at transplantation and development of grade 2–4 aGVHD was strongly related to higher NRM and lower OS. Taken together, the current study suggests that improving CsA monitoring and dose adjustments may lead to a reduction of grade 2–4 aGVHD and even decrease NRM in the RIC setting.

The main limitations of our study include its retrospective nature and the evaluation of median CsA concentrations instead of the area under the curve over time [27, 28], a much more precise monitoring strategy which, however, is difficult to implement in daily clinical practice. In conclusion, the current study suggests that low median CsA concentrations in the early posttransplant period are associated with a higher incidence of grade 2–4 aGVHD in patients receiving an allo-RIC, suggesting that maintaining high CsA blood levels in the absence of organ toxicity may translate into lower risk of grade 2–4 aGVHD and improved long-term outcomes.

## Figures and Tables

**Table 1:** Patients' characteristics.

Characteristics	Population (*n* = 156)
Median age, years (range)	52 (17–69)
Patient sex, *n* (%)	
Female	64 (41%)
Sex-mismatch, *n* (%)	
Female to male	41 (26.3%)
Diagnosis, *n* (%)	
AML/MDS	28 (18%)/17 (11%)
ALL	5 (3%)
NHL/CLL	30 (20%)/11 (7%)
HD	23 (15%)
MM	33 (21%)
CML/other CMPN	7 (5%)/2 (1%)
ECOG, *n* (%)	
0	135 (86.5%)
1	10 (6.4%)
2	11 (7.1%)
Reason for RIC, *n* (%)	
Advanced age	77 (49.4%)
Multiple prior lines of treatment and/or prior transplantation	24 (15.4%)
Comorbidities and/or ECOG >1	10 (6.4%)
More than one reason	45 (28.8%)
Disease status, *n* (%)	
Early phase	72 (46%)
Advanced phase	84 (54%)
Renal impairment before SCT, *n* (%)	
None	129 (83%)
GFR 50–60 mL/min	16 (10%)
GFR 40–50 mL/min	9 (6%)
GFR < 40mL/min	2 (1.3%)
CMV serostatus (donor and receptor negative), *n* (%)	10 (6.4 %)
Conditioning regimen, *n* (%)	
Fludarabine-melphalan	103 (65%)
Fludarabine-busulfan	53 (35%)
CD34^+^ × 10E6/kg infused, median (range)	6.7 (1.6–15.6)
GVHD prophylaxis, *n* (%)	
CsA-MTX	121 (77.6%)
CsA-MMF	35 (22.4%)
Follow-up for survivors in months: median (range)	67 (5–121)

Notes: AML: acute myeloid leukemia; MDS: myelodisplastic syndrome; ALL: acute lymphatic leukemia; NHL: non-Hodgkin lymphoma; CLL: chronic lymphoid leukemia; HD: Hodgkin disease; MM: multiple myeloma; CML: chronic myeloid leukemia; CMPN: chronic myeloproliferative neoplasms; GFR: glomerular filtration rate.

**Table 2:** Description of Csa levels during the first four weeks after stem cell transplantation.

CsA blood concentration; median (range) ng/mL	
First week	134 (10–444)
Second week	219 (54–656)
Third week	253 (53–910)
Fourth week	224 (30–699)
Patients with CsA median levels in the optimal range (200–300 ng/mL); *n* (%)	
First week	22/149 (15%)
Second week	67/153 (44%)
Third week	66/148 (45%)
Fourth week	53/123 (43%)
Patients with one or more CsA blood level(s) below 150 ng/mL; *n* (%)	
First week	124 (83%)
Second week	51 (33%)
Third week	28 (19%)
Fourth week	26 (21%)
Patients with CsA median levels lower than 150 ng/mL *n* (%)	
First week	89 (60%)
Second week	24 (16%)
Third week	16 (11%)
Fourth week	21 (17%)

Note: CsA: cyclosporine A.

**Table 3:** Incidence and characteristics of graft versus host disease.

Patients with aGVHD, *n* (%)	
Grade 1	32 (20.5%)
Grade 2	32 (20.5%)
Grade 3	18 (11.5%)
Grade 4	5 (3.2%)
Incidence of aGVHD, %, (95% CI)	
Any grade	58% (50–64%)
Grade 2–4	45% (34–50%)
Organs involved, *n* (%)	
Skin	75 (48%)
Gastrointestinal tract	45 (29%)
Liver	45 (29%)
Day onset of aGVHD, median (range)	
Any grade	43 (12–185)
Grades 2–4	42 (16–185)

Note: aGVHD: acute graft versus host disease.

**Table 4:** Univariate and multivariate analysis for aGVHD.

	Grade 2–4 aGVHD			
Variable	Univariate analysis	Multivariate analysis		
	*P*	HR	CI 95%	*P*
Sex-mismatch (female to male)	0.01	2.5	1.5–4.3	0.01
High CsA concentration				
Second week	0.02			NS
Third week	0.01	0.99	0.98-0.99	0.03
Patient sex (male)	0.01			NS

The patients with median CsA levels higher than 150 ng/mL are included in the group “high CsA concentration.”

Notes: LR: log-rank; HR: hazard ratio; NS: not statistically significant.

CMV status, disease status at SCT, age of the recipient, conditioning regimen, immunosuppressive schedule, and CsA levels during the first and fourth week after transplantation were not associated with acute GVHD in univariate analysis (*P* > 0.1).

**Table 5:** Univariate and multivariate analysis for nonrelapse mortality and overall survival.

	Nonrelapse mortality			Overall survival		
Variables	Univariate analysis	Multivariate cox regression		Univariate analysis	Multivariate cox regression	
	*P* value	HR (95% CI)	*P*	*P* value	HR (95% CI)	*P* value
Sex-mismatch (female to male)	<0.01		NS	0.02		NS
Advanced disease status	0.01	2.2 (1.3–4.3)	0.01	0.02	2.2 (1.1–4.3)	0.02
Low median CsA levels in 3rd week*	0.04		NS (0.06)	0.08		NS
Renal impairment*	0.06		NS	0.08		NS
Grade 2–4 aGVHD*	<0.01	2.5 (1.5–4.4)	0.01	<0.01	2.5 (1.4–4.4)	<0.01

Note: CsA: cyclosporine A; aGVHD: acute graft versus host disease; HR: hazard ratio; CI: confidence interval.

*Variables analyzed as time-dependent covariates.

## References

[1] Martino R, Valcárcel D, Brunet S, Sureda A, Sierra J (2008). Comparable non-relapse mortality and survival after HLA-identical sibling blood stem cell transplantation with reduced or conventional-intensity preparative regimens for high-risk myelodysplasia or acute myeloid leukemia in first remission. *Bone Marrow Transplantation*.

[2] Valcárcel D, Martino R, Caballero D (2008). Sustained remissions of high-risk acute myeloid leukemia and myelodysplastic syndrome after reduced-intensity conditioning allogeneic hematopoietic transplantation: chronic graft-versus-host disease is the strongest factor improving survival. *Journal of Clinical Oncology*.

[3] Melve GK, Ersvssr E, Kittang AO, Bruserud O (2011). The chemokine system in allogeneic stem-cell transplantation: a possible therapeutic target?. *Expert Review of Hematology*.

[4] Handschumacher RE, Harding MW, Rice J, Drugge RJ, Speicher DW (1984). Cyclophilin: a specific cytosolic binding protein for cyclosporin A. *Science*.

[5] Izumi N, Furukawa T, Sato N (2007). Risk factors for acute graft-versus-host disease after allogeneic hematopoietic stem cell transplantation: retrospective analysis of 73 patients who received cyclosporin A. *Bone Marrow Transplantation*.

[6] Malard F, Szydlo RM, Brissot E (2010). Impact of cyclosporine-A concentration on the incidence of severe acute graft-versus-host disease after allogeneic stem cell transplantation. *Biology of Blood and Marrow Transplantation*.

[7] Bontadini A (2012). HLA techniques: typing and antibody detection in the laboratory of immunogenetics. *Methods*.

[8] Delgado J, Marco A, Moreno E (2009). Reduced-intensity conditioning allogeneic hematopoietic cell transplantation using oral fludarabine as part of the conditioning regimen. *Cytotherapy*.

[9] Piñana JL, Valcárcel D, Fernández-Avilés F (2010). MTX or mycophenolate mofetil with CsA as GVHD prophylaxis after reduced-intensity conditioning PBSCT from HLA-identical siblings. *Bone Marrow Transplantation*.

[10] Andrews DJ, Cramb R (2002). Cyclosporin: revisions in monitoring guidelines and review of current analytical methods. *Annals of Clinical Biochemistry*.

[11] Przepiorka D, Weisdorf D, Martin P (1995). 1994 consensus conference on acute GVHD grading. *Bone Marrow Transplantation*.

[12] Martino R, Subirà M (2002). Invasive fungal infections in hematology: new trends. *Annals of Hematology*.

[13] Piñana JL, Martino R, Barba P (2010). Cytomegalovirus infection and disease after reduced intensity conditioning allogeneic stem cell transplantation: single-centre experience. *Bone Marrow Transplantation*.

[14] Kanda Y, Hyo R, Yamashita T (2006). Effect of blood cyclosporine concentration on the outcome of hematopoietec stem cell transplantation from an HLA-matched sibling donor. *American Journal of Hematology*.

[15] Klein JP, Rizzo JD, Zhang M-J, Keiding N (2001). Statistical methods for the analysis and presentation of the results of bone marrow transplants—part I: unadjusted analysis. *Bone Marrow Transplantation*.

[16] Klein JP, Rizzo JD, Zhang M-J, Keiding N (2001). Statistical methods for the analysis and presentation of the results of bone marrow transplants—part 2: regression modeling. *Bone Marrow Transplantation*.

[17] Kaplan EL, Meier P (1958). Nonparametric estimation from incomplete observations. *Journal of the American Statistical Association*.

[18] Song M-K, Chung J-S, Seol Y-M (2009). Influence of lactate dehydrogenase and cyclosporine a level on the incidence of acute graft-versus-host disease after allogeneic stem cell transplantation. *Journal of Korean Medical Science*.

[19] Martin P, Bleyzac N, Souillet G (2003). Relationship between CsA trough blood concentration and severity of acute graft-versus-host disease after paediatric stem cell transplantation from matched-sibling or unrelated donors. *Bone Marrow Transplantation*.

[20] Lanino E, Rondelli R, Locatelli F (2009). Early (day −7) versus conventional (day −1) inception of cyclosporine-A for graft-versus-host disease prophylaxis after unrelated donor hematopoietic stem cell transplantation in children. Long-term results of an AIEOP prospective, randomized study. *Biology of Blood and Marrow Transplantation*.

[21] Piñana JL, Valcárcel D, Martino R (2009). Study of kidney function impairment after reduced-intensity conditioning allogeneic hematopoietic stem cell transplantation. A single-center experience. *Biology of Blood and Marrow Transplantation*.

[22] Bennett WM, Pulliam JP (1983). Cyclosporine nephrotoxicity. *Annals of Internal Medicine*.

[23] Parikh CR, Sandmaier BM, Storb RF (2004). Acute renal failure after nonmyeloablative hematopoietic cell transplantation. *Journal of the American Society of Nephrology*.

[24] Kersting S, Dorp SV, Theobald M, Verdonck LF (2008). Acute renal failure after nonmyeloablative stem cell transplantation in adults. *Biology of Blood and Marrow Transplantation*.

[25] Satwani P, Harrison L, Morris E, del Toro G, Cairo MS (2005). Reduced-intensity allogeneic stem cell transplantation in adults and children with malignant and nonmalignant diseases: end of the beginning and future challenges. *Biology of Blood and Marrow Transplantation*.

[26] Mori T, Kato J, Shimizu T (2012). Effect of early posttransplantation tacrolimus concentration on the development of acute graft-versus-host disease after allogeneic hematopoietic stem cell transplantation from unrelated donors. *Biology of Blood and Marrow Transplantation*.

[27] Jin M, Seto W, Taylor T, Saunders EF, Doyle J, Dupuis LL (2008). Determination of initial i.v. CYA dosage to achieve target AUC values in pediatric hematopoietic stem cell transplant patients. *Bone Marrow Transplantation*.

[28] Duncan N, Arrazi J, Nagra S, Cook M, Thomson AH, Craddock C (2010). Prediction of intravenous cyclosporine area under the concentration-time curve after allogeneic stem cell transplantation. *Therapeutic Drug Monitoring*.

